# Role of Extracellular Vesicles in Hematological Malignancies

**DOI:** 10.1155/2015/821613

**Published:** 2015-10-25

**Authors:** Stefania Raimondo, Chiara Corrado, Lavinia Raimondi, Giacomo De Leo, Riccardo Alessandro

**Affiliations:** ^1^Dipartimento di Biopatologia e Biotecnologie Mediche, Università degli Studi di Palermo, Sezione di Biologia e Genetica, Via Divisi 83, 90133 Palermo, Italy; ^2^Laboratorio di Ingegneria Tissutale-Piattaforme Innovative per l'Ingegneria Tessutale (PON01-00829), Istituto Ortopedico Rizzoli, 90133 Palermo, Italy; ^3^Institute of Biomedicine and Molecular Immunology (IBIM), National Research Council of Italy, 90146 Palermo, Italy

## Abstract

In recent years the role of tumor microenvironment in the progression of hematological malignancies has been widely recognized. Recent studies have focused on how cancer cells communicate within the microenvironment. Among several factors (cytokines, growth factors, and ECM molecules), a key role has been attributed to extracellular vesicles (EV), released from different cell types. EV (microvesicles and exosomes) may affect stroma remodeling, host cell functions, and tumor angiogenesis by inducing gene expression modulation in target cells, thus promoting cancer progression and metastasis. Microvesicles and exosomes can be recovered from the blood and other body fluids of cancer patients and contain and deliver genetic and proteomic contents that reflect the cell of origin, thus constituting a source of new predictive biomarkers involved in cancer development and serving as possible targets for therapies. Moreover, due to their specific cell-tropism and bioavailability, EV can be considered natural vehicles suitable for drug delivery. Here we will discuss the recent advances in the field of EV as actors in hematological cancer progression, pointing out the role of these vesicles in the tumor-host interplay and in their use as biomarkers for hematological malignancies.

## 1. Introduction

Cell-to-cell communication is necessary in order to maintain a social and functional order among different cell types within tissues. A number of intercellular communication mechanisms mediated, for example, by soluble factors, extracellular matrix components, ion channels, tunneling nanotubules, and extracellular vesicles (EV) have been described [1].

EV are plasma membrane fragments that include, among several others, microvesicles (MV) and exosomes [2]. MV enclose a heterogeneous population of vesicles with a size greater than 100 nm in diameter and are generated by direct budding off from the plasma membrane; Umezu and colleagues; miR-135b; NF-*κ*B [3]. The rate of release of MV is generally low, except for cancer cells, which show an intense surface activity and seem to release them constitutively. Exosomes are vesicles of size ranging from 30 to 100 nm in diameter and are released into the extracellular compartment when the multivesicular bodies (MVB) fuse with the plasma membrane [3]. The secretion of exosomes can be spontaneous or induced, for example, by cell surface receptor activation [2–4]. The mechanisms of assembly and sorting of exosomes are not well defined, but several molecules have been shown to regulate this process, such as RAB11, RAB27, RAB35, and syndecan-syntenin-ALIX [5–8]. Moreover the ESCRT (endosomal sorting complex required for transport) member TSG101 (tumor susceptibility gene 101) and the tetraspanin CD63, which is enriched in specific plasma membrane domains involved in microvesicle budding, have both been described as involved in exosome formation [9]. Recently, the consideration of the tetraspanin CD63 as a specific exosomal marker has been reevaluated, since this protein has also been found in other EV subtypes [10].

The molecular composition of EV varies depending on the type and functional state of the producing cell. During cancer progression, exosomes, released from malignant cells, are enriched in tumor antigens: for example, exosomes isolated from ovarian or breast cancer ascites contain tumor specific antigens HER2/NEU, while MART1 is found in nanovesicles recovered from serum of patients with melanoma [11].

In addition to proteins and specific lipids, coding and noncoding RNAs and DNA may be present in EV [1, 12]. The molecular components found in EV could be transferred from one cell to another by endocytosis or fusion with the recipient cell [13, 14]. Of biological interest, the transferred components are functional in target cells and affect the phenotype of these cells by modulating gene expression [1, 12, 15, 16]. Thus, EV-mediated intercellular communication includes the binding of cell-surface receptors on target cells, followed by induction of cell signaling pathways, transfer and translation of mRNAs, transfer of microRNAs (miRNAs), silencing of mRNA targets, transfer of proteins, and activation of functions. Moreover, after their release into the circulation, EV-dependent signaling can occur not only locally, but also in a paracrine and systemic manner [17, 18]. Even if microvesiculation is a mechanism occurring in all eukaryotic cells, the amount of EV in the blood is increased in several pathological conditions, including Alzheimer's disease, Crohn's, immunological disorders, and cancer [19]. EV have been shown to support tumor invasiveness and metastasis, as well as enhancing angiogenesis. In recent years the scientific community has focused its attention on the specific properties of the tumor microenvironment and how these depend on altered intercellular communication between malignant cells and nonmalignant cells of the host. A number of studies have focused on the role played by cancer-cell-derived EV. For example, the transformation of fibroblasts and epithelial cells upon uptake of glioblastoma- and breast-cancer-cell-derived EV was partly dependent on the transfer of fibronectin and transglutaminase [20]. Cancer cells induce adaptive mechanisms that rely on phenotypic modulation of stromal cells in the neighboring tissue and the recruitment of bone-marrow-derived progenitors from the circulation. This adaptive remodeling of the tumor microenvironment, involving the induction of a large number of effector molecules, ultimately serves to promote the survival and dissemination of malignant cells. EV can modulate the stroma, thus promoting cancer progression and metastasis. Peinado et al. have recently shown that cancer-derived exosomes modulate the crosstalk between malignant cells and the bone marrow (BM) microenvironment. They reported, for the first time, that metastatic melanoma cells release exosomes that are able to “educate” BM progenitors, thereby inducing their mobilization, which supports tumor vasculogenesis, invasion, and metastasis, through the activation of the MET receptor tyrosine kinase. They found that the MET-activated signaling proteins are expressed in highly metastatic melanoma derived exosomes and that the transfer of the exosomal receptor tyrosine kinase MET from tumor-derived exosomes to BM progenitor cells promotes the metastatic process* in vivo*. These results suggest that BM cells retain the educated phenotype after engraftment into a new host [21]. A recent study by Melo et al. presents new and significant data on the role of exosome-shuttled miRNAs in cancer progression. In particular, the authors show that exosomes derived from breast cancer cells, and sera from patients, contain DICER and process pre-miRNAs into mature miRNAs, thus modulating the recipient cell phenotype. This study points out a new role for exosomes, which can be considered not only a shuttle for miRNAs, but also as machinery for miRNA biogenesis [22].

Hypoxia is a common feature of the microenvironment of malignant tumors [23, 24] and is associated with tumor aggression and invasiveness. These effects are mediated, for example, by hypoxic regulation of cytokine release [25] and regulation of tumor suppressors and oncogenes [26]. Several reports have shown a correlation between hypoxia, tumor progression, and exosome release. For example, King et al. found a hypoxic enhancement of exosome release by breast cancer cells [27]. Moreover, exosomes reflect the hypoxic status of tumor cells by mediating microvascular endothelial cell (EC) migration and vasculogenesis [28, 29].

Here we will focus on the role of EV in hematological malignancies and in particular on (i) the role of EV in the crosstalk between cancer cells and the BM microenvironment; (ii) the role of cancer-cell-secreted EV in angiogenesis; and (iii) the role of EV as biomarkers of hematologic malignancies and in mechanisms of drug resistance.

## 2. EV in the Crosstalk with the BM Microenvironment

The importance of tumor microenvironment for cancer progression has, in recent years, been widely recognized. The microenvironment provides crucial signaling to maintain tissue architecture, inhibit cell growth, and modulate differentiated phenotypes. On the other hand, incorrect signals from the microenvironment may lead to destabilization of tissue homeostasis and to the initiation/promotion of normal cells to malignancy. Furthermore, the interaction of cancer cells with their stromal microenvironment overcomes the physiological barrier function of stromal cells and may also modulate the invasive and metastatic phenotype of the cancer cells, as well as angiogenesis [30]. Several studies in the last five years have indicated that EV are important components of the tumor microenvironment and, mediating cell-cell communication, are currently considered one of the contributors to tumor progression and metastasis [31]. Moreover, EV have been found to contribute not only to primary tumor growth, but also to the recruitment of microenvironment resident cells such as ECs or leukocytes (e.g., macrophages, dendritic cells, and T- and B-cells) [17, 32, 33]. Therefore, coevolution of the tumor microenvironment with primary tumor cells is promoted by the crosstalk of different cell types in a tumor EV-dependent manner [34].

**Figure 1:**
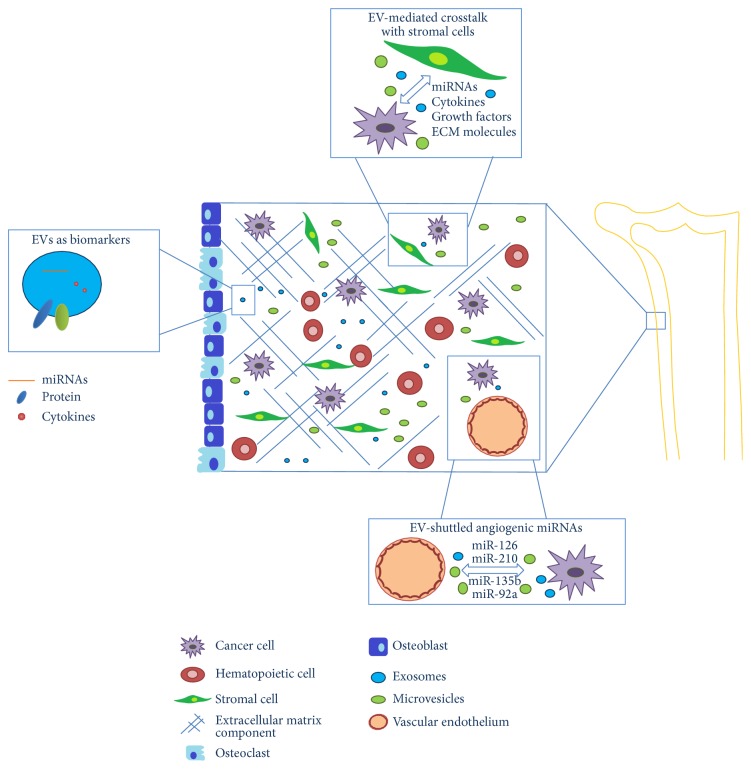
The bone marrow niche and the major constituents relevant to hematological cancer progression: extracellular vesicles (exosomes and microvesicles) are integral part of tumor microenvironment, contributing to the development of a suitable niche for hematological cancer development. Extracellular vesicles released by different cell types allow the bidirectional communication among cancer cells and normal host cells.

BM is the specific microenvironment of hematological disease and is composed of a dynamic network of stromal cells and soluble factors, such as growth factors and cytokines, thus providing a permissive environment for leukemogenesis and cancer progression (Figure 1). In acute myeloid leukemia (AML), exosomes released from leukemia cells alter the proliferative and migratory responses of cocultured stromal and hematopoietic progenitor cells, helping to explain how the microenvironmental niche becomes reprogrammed during invasion of the BM by AML cells. Specifically, Huan et al. showed the presence of transcripts with prognostic relevance in AML, human CXCR4 and IGF-IR (insulin-like growth factor-I receptor) mRNA, in murine BM stromal cells, thus demonstrating that AML exosomes transfer leukemia-derived mRNAs to BM stromal cells. Moreover, they found that exosomal transfer of IGF-IR can modulate proliferative signaling in bystander cells and can promote expression of VEGF, with the final establishment of conditions that contribute to leukemic spread [35]. We recently provided data showing that chronic-myeloid-leukemia- (CML-) derived exosomes are able to stimulate bone marrow stromal cells (BMSC) to release IL8, which acts as an* in vitro* and* in vivo* prosurvival factor for CML cells. The inhibition of IL8 receptors, using SB225002, was able to abrogate the IL8- driven CML cell survival* in vitro* as well as the growth of CML xenograft* in vivo*, thus indicating a key role of CXCLl8/CXCR1-2 signaling in the growth of CML cells [36]. It is conceivable that IL8 secreted by the BM and ECs under the stimulation of CML exosomes may modulate both myeloid malignant cells and the BM cellular compartment, thus generating a paracrine loop between hematopoietic malignant cells and resident cells. Our unpublished data further shows that the CML-exosome-mediated release of IL8 by BMSC relies on the activation of the EGF receptor pathway. On the other hand, IL8 is able to activate, in CML cells, an AXL mediated pathway that could be responsible for leukemia cell survival (unpublished data). Ghosh et al. found that EV isolated from plasma of B-cell chronic lymphocytic leukemia (CLL) patients can interact and modulate BMSC, thus providing a “*homing and nurturing*” environment for CLL B cells. In particular, they demonstrated that CLL-EV can maintain the AKT/mTOR/p70S6K/HIF-1*α* axis in a sustained state of activation and can potentially modulate the AKT/GSK3*β* or AKT/*β*-catenin signaling pathways. This leads to the establishment of a tumor microenvironment that favors disease progression [37].

Cell-to-cell communication can also occur between leukemia cells and normal neutrophils, thus providing a mechanism for tumor development. Cai's group found that Bcr/Abl hybrid gene, involved in the pathogenesis of CML, could be transferred through K562 EV to normal neutrophils. Incubation of neutrophils with K562-EV for 24 hours resulted in the expression of the Bcr/Abl hybrid gene in 20% of the cells [38]. Moreover, the same group showed that injection via tail vein of K562 EV into Sprague-Dawley (SD) rats or NOD/SCID mice caused several symptoms of CML in the animals, such as weakness, loss of weight, splenomegaly, and neutrophilia, but reduced neutrophil phagocytic activity. Disease development was accompanied with* de novo* transcription, as well as protein synthesis of BCR/ABL* in vivo*, demonstrating that the transfer of the Bcr/Abl gene from CML-derived EV to neutrophils may promote* in vivo* transformation of normal cells [39]. During tumor development, neoplastic cells actively recruit cells of the immune system, which may provide an immunosuppressive and growth-promoting compartment [40]. Recently, numerous studies have shown that microenvironmental stressors such as low pH, heat, and oxidative stress modulate the molecular composition of EV [41]. For example, leukemia/lymphoma T- and B-cells under a thermal and oxidative stress release exosomes enriched in Natural Killer Group 2, member D (NKG2D) ligands, which abrogate NKG2D-mediated NK-cell cytotoxicity and, thus, may contribute to the immune evasion of leukemia/lymphoma cells [42]. Stromal cells, similarly to cancer cells, can respond to stress-related conditions within the tumor microenvironment by secretion of EV. For example, mesenchymal stem cells stimulated by hypoxia were shown to release MV with angiogenic potential [43]. Roccaro et al. reported that multiple-myeloma-BM-mesenchymal-stromal-cell- (MM-BMSC-) derived exosomes played a role in multiple myeloma (MM) disease progression* in vivo*. In particular, they showed that BMSC transfer exosomes containing miR-15a into MM cells, inducing their proliferation and survival. Importantly, while MM-BM-MSC–derived exosomes promoted MM tumor growth, normal BM-MSC exosomes inhibited the growth of MM cells. These observations suggest the establishment of paracrine growth circuits between BM-MSC and clonal plasma cells and that the BM niche, educated by tumor exosomes, provides an optimal substrate for MM cell localization and growth [44]. The data reported here underscore the importance of the tumor microenvironment for cancer progression. In particular, in the context of hematological malignancies, we found examples of the role of EV released by cells of hematological malignancies in the crosstalk with BM resident cells.

## 3. EV in Angiogenesis

The tumor vasculature is an important component of the tumor microenvironment. Tumors are endowed with angiogenic-inducing capability, and their growth, invasion, and metastasis are angiogenesis-dependent [45]. Over the last few years, many studies have focused their attention on the role of these vesicles in modulating angiogenic processes [46]. EV possess the ability to modulate tumor angiogenesis, depending on their origin and composition, by inducing changes in target cells or by delivering angiogenic proteins or miRNAs that can stimulate EC function. Here we will focus on the role of EV in hematological disorders. Increased angiogenesis has been observed in hematologic disorders, including acute and CML, acute and chronic lymphocytic leukemia, MM, and lymphomas [47–50]. BM microvessel density, a measure of tumor angiogenesis, is greater in patients with advanced myelodysplastic syndromes compared with normal individuals, thus confirming a central role of the process in these disorders [51]. In recent years, we and other groups have focused research on the role of exosomes derived from chronic myelogenous leukemia cells in promoting angiogenesis. Taverna et al. showed that exosomes released from CML cells directly affect ECs by modulating the process of neovascularization, both* in vitro* and* in vivo*, by inducing in ECs the release of proangiogenic cytokines, such as Interleukin-8 [52]. We have also shown that imatinib-resistant CML cells release exosomes with proangiogenic abilities, suggesting that the modulation of their function could be considered for new approaches in CML treatment [53]. Mineo et al. reported that exosomes released by K562 CML cells are internalized by ECs during tubular differentiation on Matrigel and are transferred to neighboring cells through the formation of nanotubular structures connecting the cells. Furthermore, the authors showed that these exosomes stimulate tube formation in ECs through SRC activation [54]. It is now widely known that EV contain miRNAs and that they are delivered in the tumor microenvironment [12, 55]. To date, many angiogenic miRNAs have been identified, and several have been shown to play important roles in exosome-mediated modulation of angiogenesis. Taverna et al. recently found that miR-126, a miRNA involved in angiogenesis, was expressed 6-fold more in LAMA84 exosomes compared with the parental cells. The exosomal transfer of miR-126 to ECs directly targeted the 3′ UTR of Cxcl12 and Vcam1 mRNA, thus modulating adhesive and migratory abilities of CML cells [56]. Other groups have found that exosomes released from K562 cells contain specific miRNAs, which enhanced EC migration and tube formation [57, 58]. Umezu et al. reported that miR-92 derived from K562 cells was transferred to ECs through exosomes, thus promoting endothelial migration and tube formation [57]. It is well established that hypoxia plays a role in boosting tumor angiogenesis. Many studies have shown that the exposure of human cancer cells to hypoxia augments microvesicle shedding in order to modify their microenvironment to facilitate tumor angiogenesis and metastasis [59]. Moreover, microarray and proteomic studies have highlighted the increase of many proangiogenic factors in EV released after hypoxic stimuli [58, 60]. Tadokoro and colleagues reported that the hypoxic human leukemia cell line K562 secretes exosomal miRNAs, which enhances tube formation in human umbilical vein ECs due to inhibition of the receptor tyrosine kinase ligand EPHRIN-A3. In particular, they found that hypoxic exosomes contained higher levels of miR-210, indicating a difference between normoxic and hypoxic exosomes [58]. In AML, the release of EV has been described* in vitro* as well as in the sera of patients affected with the disease. Kurre's group has shown that AML exosome trafficking alters the angiogenic responses of cocultured stromal and hematopoietic progenitor cell lines, thus influencing the invasion of the BM [35]. BM angiogenesis also plays an important role in the pathogenesis and progression of MM. The tumor-host interplay, driven by EV, in MM has been recently established. Liu et al. reported, for the first time, that myeloma RPMI 8226 cells can secrete MV harboring oncogenic CD138, a specific type of angiogenic regulator, and the incorporation of the MM-MVs into ECs leads to the reprogramming of the ECs. Specifically, exosome stimulation promotes EC proliferation and the invasion and the secretion of the proangiogenic factors IL6 and VEGF [61]. Recently, Umezu and colleagues have established an* in vitro* system of hypoxic-resistant multiple myeloma cells (HR-MM), which serves as a model of therapy-resistant MM cells. The authors showed that the amount of exosomes from HR-MM cells was significantly greater than that of parental cells and that specific miRNAs were present. They found that hypoxia-driven accelerated tube formation is attributable to exosomal miR-135b, an oncogenic miRNA shed from HR-MM cells. Specifically, they found that miR-135b directly targets and inhibits FIH-1, which is an asparaginyl hydroxylase enzyme binding to HIF-1*α*, thus inhibiting its transactivation. In this way, exosomal miR-135b accelerated HIF-1 transcriptional activity via inhibition of FIH-1 [60]. Together, these data highlight the crucial role of EV in tumor-host communication, thus contributing to the progression and dissemination of cancer cells through the modulation of angiogenic processes.

## 4. EV as Biomarkers of Hematological Malignancies and Their Role in Mechanisms of Drug Resistance

The identification of new biomarkers differentially expressed in cell populations, as well as in patients, is of primary importance in the detection, diagnosis, and prognosis of cancer. Biologically active exosomes isolated from human plasma can be proposed as new and intriguing biological markers [62]. Because EV (both MV and exosomes) contain genetic and proteomic contents that reflect the cell of origin, it is possible to detect tumor-specific material in EV secreted by cancer cells.

It has been clearly shown that the release of both MV and exosomes is dramatically enhanced during cancer progression. Moreover, EV can be recovered from body fluids and the circulation of cancer patients, thereby constituting a source of new predictive biomarkers involved in cancer development and potentially serving as an information-rich prognostic [63–65]. Working as shuttles, EV can protect their cargo from nucleases and proteases, increasing the biomarker half-life and potentially facilitating sample integrity and downstream analyses [66]. EV-associated cancer biomarkers have been extensively described in a great number of solid cancers, such as prostate cancer, melanoma, and lung cancer [21, 67–70]. Furthermore, in patients affected with melanoma and other solid tumors, total protein levels of exosome fractions isolated from plasma were reported to reflect disease stage, tumor burden, response to therapy, and survival [21, 71, 72]. However, EV derived from hematological cancer cells have been less studied than those derived from solid tumor cells. An interesting study showed that in AML, exosomal protein levels may reflect the extent of disease and correlate with its relapse after therapy. Large differences were noted in exosomal protein levels among patients upon AML diagnosis, and the authors suggested that low exosomal protein levels may be predictive of long-term disease survival. In addition, change in exosomal protein levels may reflect response to chemotherapy, and the exosomal profile analysis may detect residual leukemia cells in patients considered in complete remission. More specifically, TGF-*β*1 levels were analyzed and compared among patient cohorts, and the changes in exosome-associated TGF-*β*1 levels were evaluated in relation to the therapy given to AML patients. The authors found that the TGF-*β*1 levels upon AML diagnosis were higher than those in exosomes of normal controls. Following chemotherapy treatment, TGF-*β*1 levels were significantly reduced, while patients in long-term complete remission had low exosomal TGF-*β*1 levels. The authors suggested that change in exosomal TGF-*β*1 levels may reflect responses to chemotherapy. According to the authors, these data reinforced the relevant role of AML-derived exosomes as potential diagnostic or prognostic biomarkers [71].

EV shuttle diverse RNA species, including mRNAs and miRNAs, to recipient cells, and affect the metabolism of target cells. In addition, several data have shown that miRNA content from their originating cancer cells is similar to that found in circulating exosomes [73]. Exosomes derived from both AML and CML cells were enriched for several coding and noncoding RNAs relevant to both cancer prognosis and treatment, as well as to the leukemic niche function. Still, CML-derived exosomes are characterized by selectively expressed miRNAs and plausibly suggested as possible future biomarkers [35]. A recent study identified miR-155 as a useful biomarker in individuals with monoclonal B-cell lymphocytosis and in patients with B chronic lymphocytic leukemia. The authors found higher miR-155 levels expression in patients who failed to respond to chemotherapy compared with those who experienced complete response. Furthermore, the authors identified miR-155 in circulating MV from both individuals with monoclonal B-cell lymphocytosis and patients with chronic lymphocytic leukemia [74].

Because the residual disease in patients considered in complete remission is difficult to investigate with conventional methods, the need to elaborate alternative tests for detecting residual disease will become more pressing in future studies. At present, miRNA-based clinical trials involving exosomes have not been initiated because an improved characterization of these carriers and their cargos in normal and disease models is still needed [41].

The development of chemotherapeutic resistance is one of the major factors that contribute to cancer mortality. Though a combination of molecular mechanisms is responsible for drug resistance, the role played by EV in modulating the acquisition of the chemoresistant phenotype is increasingly emerging [41, 75–79]. The effect of BMSC-derived exosomes on the survival and drug resistance of MM cells, using both a murine model and human MM samples, has been investigated in MM. The authors found drug resistance to bortezomib in MM cells treated with BMSC-derived exosomes, as well as the activation of several pathways related to drug resistance and cell survival, such as NOTCH1, STAT3, NF-*κ*B, and AKT [80]. In another study, Aung et al. found, both* in vitro* and* in vivo*, a strong exosome production and release from aggressive B-cell lymphoma cells. Such lymphoma-derived exosomes carried the protein CD20, exposed in the membrane, able to intercept rituximab, and thus allowing lymphoma cells to escape from humoral immunotherapy [81]. Because exosomal CD20 is a decoy target for rituximab, the authors suggested that the drug sequestered by circulating exosomes may reduce the efficacy of pharmacological treatment [81].

Cancer cells exposed to chemotherapeutic agents are able to expel drugs in extracellular compartments using specialized transporters of the multidrug resistant ATP binding-cassette transporter (ABC transporter). Recent studies have indicated that leukemia cells express ABCA3 transporters and that their expression is correlated with decreased susceptibility to cytostatic therapy. ABCA3 is localized inside the limiting membranes of multivesicular bodies in which drugs are efficiently sequestered [81–84]. It is now clear that exosomes from different malignant cells carry ABC transporters, and some studies have shown that drugs may also be expelled from the cells through exosomal mechanisms [41, 81, 85, 86]. Finally, immunotherapy studies in aggressive B-cell lymphoma demonstrated for the first time that exosomal evasion of humoral immunotherapy was modulated by ABCA3 [81].

Collectively, these studies provide evidence that EV in hematological malignancies play an important role in cancer drug resistance, thus influencing response to therapy and promoting cancer progression. Advances in the understanding of cancer-derived EV biology, as well as in the development of new sophisticated technologies aimed at isolating and characterizing EV, will be clinically relevant to the identification of new prognostic or predictive biomarkers in hematological malignancies.

## 5. Concluding Remarks

EV have been widely recognized as important actors in cell-cell communication by delivering messages among different cell types. This finding has opened new questions in the field of cancer research, allowing a new interpretation of the tumor microenvironment and of targeted therapies. Here we focused on the role of EV in hematological malignancies, reporting evidence of the importance of these vesicles in modulating cancer properties. Furthermore, EV components can now be considered biomarkers in hematological neoplasia, thus providing important findings for the early diagnosis of these malignancies and the design of alternative approaches to cancer therapy.
